# Association of Thrombomodulin Gene Polymorphism (C1418T) With Coronary Artery Disease in Pakistani Population

**DOI:** 10.12669/pjms.343.14864

**Published:** 2018

**Authors:** Muhammad Akbar Mughal, Muhammad Saleh Soomro, Syed Muhammad Ashraf Jahangeer AlSaani, Saba Shahid, Shariq Ahmed

**Affiliations:** 1 Dr. Muhammad Akbar Mughal, MBBS, MPhil. Associate Professor, Department of Physiology, Karachi Medical & Dental College, Karachi, Pakistan; 2 Prof. Dr. Muhammad Saleh Soomro, Ph.D. Chairman. Department of Physiology, Baqai Medical University, Karachi, Pakistan; 3 Dr. Syed Muhammad Ashraf Jahangeer AlSaani, MBBS, FCPS. Assistant Professor, Dow University of Health Sciences (DUHS), Karachi, Pakistan; 4 Dr. Saba Shahid, Ph.D. Assistant Professor, Genomics and Molecular Pathology Lab, National Institute of Blood Disease (NIBD), Karachi, Pakistan.; 5 Shariq Ahmed, MSc, Clinical Scientist/Lab. Supervisor, Genomics and Molecular Pathology Lab, National Institute of Blood Disease (NIBD), Karachi, Pakistan

**Keywords:** Thrombomodulin (TM), Genetic polymorphism, C1418T, Coronary Artery Disease (CAD)

## Abstract

**Objectives:**

To find out the association between Thrombomodulin gene polymorphism (C1418T) with coronary artery disease in population of Karachi, Pakistan.

**Methods:**

This case-control study was conducted in Tabba Heart Institute in collaboration with the National Institute of Blood Diseases, Karachi. We compared C/T dimorphism in 92 cases with 90 control subjects by allele-specific amplification. The results of PCR were confirmed by Gene sequencing. All the laboratory methods were strictly in compliance with the international standards. All variables that were either statistically significant in the univariate analyses or potentially important with respect to prevention or biologically relevant variables were included in logistic-regression analyses. Potential confounding was assessed with the use of multivariate models adjusted for participant’s characteristics and other major risk factors for coronary artery disease. All reported p values are two-tailed, with statistical significance at p value < 0.05.

**Results:**

The frequency of CC, C/T and TT genotype was 81 (90%), 6 (6.7%) 3 (3.3%) in controls and 67 (72.8%), 20 (21.7%) and 5 (5.4%) in cases respectively. In cases group the CT/TT genotypes were found to be significantly highly represented among the patients with coronary artery diseases when compared with control group (p-value 0.009).

**Conclusion:**

TM C1418T polymorphism emerges as a risk marker in Coronary Artery Disease patients in the population of Karachi, Pakistan.

## INTRODUCTION

Coronary Artery Disease (CAD) is the most common type of heart disease caused by the narrowing or obstruction of the coronary arteries resulting from atherosclerosis, which is characterized by deposition of cholesterol plaque in the tunica intima of vessel wall.^1^ Despite that there are several conventional coronary risk factors, such as diabetes mellitus, hypertension, smoking, alcohol intake, family history, and obesity that played an important role in the development of CAD, there is an increased awareness of the role of polymorphic variants of genes that emerge as a risk factors in the incidence of CAD.^2^ The Thrombomodulin (TM) gene is one of them.^3^ It is present on chromosome 20p11.2, possess a single exon and devoid of introns. This glycoprotein consists of 557 Amino Acids (AA) (60,300 Dalton) and expressed primarily on the luminal surface of vascular endothelium. The molecular sequence of TM gene is organized into six domains which are N-terminal lectin like domain (amino acid 1-154), a hydrophobic region (amino acid 155-222), six epidermal growth factors (EGF) like repeats (amino acid 223-462), a region rich in threonine and serine (amino acid463-497), a transmembrane domain (amino acid 498-521) and a short cytoplasmic tail (amino acid 522-557).^4^ A single nucleotide polymorphism at position C.1418 C>T of TM gene causes transition of cytosine (C) to thymidine (T) that results in substitution alanine (A) to valine (V) at protein position 455 (ala 455 Val) of the TM gene.^5^ This variation of amino acid is present on 6^th^ EGF region of the TM gene.^6^,^7^ It is considered as an important vasoprotective and thrombo resistant molecule. It forms a complex with thrombin (TM-thrombin 1:1 complex) and activates Protein C. The Activated Protein C (APC) now degrades factor Va and factor VIIIa, in the coagulation pathway.^6^ Moreover binding of thrombin with thrombomodulin results in loss of all its procoagulant activities such as conversion of fibrinogen to fibrin and activation of factor V and VIII.^8^

Thus, it acts as an important physiological anticoagulant and its deficiency leads to excessive thrombus formation. Several studies suggested that its dysfunction plays an important role in the pathogenesis of CAD.^9^

Studies conducted in different parts of the world show diverse relationship between TM gene polymorphism & CAD. Few studies have showed a positive association between TM gene variants and risk of CAD^10^, whereas others studies didn’t find any association of this variant with CAD.^11^,^12^ Therefore the aim of present study was to find out the association between thrombomodulin gene polymorphism (C1418T) with coronary artery disease in the population of Pakistan. To the best of our knowledge this is the first study to be conducted in this region that showed association between TM gene variant and CAD.

## METHODS

### Subject selection

This case control study was conducted in Tabba Heart Institute (THI), Karachi, Pakistan in collaboration with National Institute of Blood Disease (NIBD), Karachi, Pakistan. A total of 182 participants were selected for the study. Subjects selected were aged 19 years and above. These includes Coronary Artery Disease (CAD) group (n=92) and control group (n=90). The presence of CAD was confirmed on ECG, Trop-I and angiography and in some patients also by additional measures such as echocardiography, Myocardial Perfusion Scan (MPS) and CT angiography. CAD were classified as >70 obstruction of at least one of the major coronary artery i.e. Right Coronary Artery (RCA), circumflex artery and Left Anterior Descending (LAD) and 50% obstruction in left main artery. In this un-matched case-control study, both the cases and control groups were selected from the representative population. The mean age of cases was 59.24 SD 9.78 and control group was 36.06 SD 16.83. Patients with history of acute infections, malignancies, liver and renal diseases were excluded from the study. Informed consents were obtained from each participant and study protocol was well explained prior to the study. The data regarding conventional risk factors such as the history of hypertension, diabetes mellitus, smoking, obesity and family history were collected from all the participants. CAD group were selected by consecutive selection of the patients that underwent coronary angiography in the hospital, whereas the control group were selected from general population. This study was approved by Ethical Review Committee of the Tabba Heart Institute.

Study participants were defined as hypertensive if the blood pressure is >140/90 on two or more occasions or if they already are being treated with antihypertensive drugs. While the diabetics were defined if the fasting blood sugar is >125 mg/dl on two or more occasions or were already on drugs or insulin. Body mass index was categorized as follows: underweight <18.5, normal weight 18.5-24.9, overweight 25-29.9, and obese ≥30. A positive family history was defined as the presence of at least one 1^st^ or two 2^nd^ degree relatives with a history of CAD. Smoking status was identified on the basis of history.

### Blood collection and DNA extraction

5 ml of whole blood was obtained in a purple top vacutainers (containing ethylene diamine tetra acetic acid). The tubes were then placed at 4ºC till the extraction of DNA; next the DNA was extracted (using QIAamp DNA blood mini kit). In the next step PCR was run and the bands were obtained. The results of PCR were confirmed on gene sequencing. Lipid profiles were measured by Enzyme-Linked Immunosorbent Assay (ELISA).

### Genotyping of C1418T Polymorphism of TM gene

Allele-specific amplification was used for identification of single nucleotide polymorphism. The allele-specific primers that were used for the amplification of 384 base pairs of TM 1418 polymorphism are: The forward primer used for amplification of TM 1418 allele C is: TM-Ala (5’-GGGCCCGACTCGGCCCTTGC-3’), whereas the forward primer used for amplification of TM 1418 allele T is: TM-Val (5’-GGGCCCGACTCGGCCCTTGT- 3’) and the same reverse primer that is used for the amplification of TM 1418 allele:

TM-R (5’-GGGGTGAGGAGGCACAGGCTC- 3’). The sequencing primers that were used for the amplification of 935 base pairs of TM Gene are:

SF primer: (5’-TCCCCTCGGCTTACAGCTAA-3’)

SR primer: (5’-GGCACAGGCTCGCGATGGA-3’).

The PCR reaction was performed in a thermal cycler using 200 µl thin walled PCR tubes. The total reaction volume is 25 µl, were taken in each tube and comprised of: 100 ng of genomic DNA, 0.4 µM of each primer (TM-Ala and TM-R) or (TM- Val and TM-R), 2 µM of dNTPs, 1.5 mM of MgCl2, 1.25 units of Taq DNA polymerase, PCR buffer and 10 X cetos buffer.

Initially the reaction was heated for five minutes at 94 °C followed by 35 cycles of PCR with denaturation at 94°C for 30 seconds, annealing at 61°C for 60 seconds, and extension at 72°C for 30 seconds, with an additional extension cycle at 72°C for five minutes. The results of PCR reactions were run on a 2% agarose gel with ethidium bromide staining. 03 randomly selected samples were further analyzed by direct sequencing of amplified product.

### Statistical analysis

A statistical analysis plan was formulated before undertaking the study. Statistical analyses were performed using IBM SPSS statistics (IBM Corp). Univariate analyses were performed with chi-square test and student t-test where applicable. Frequency distribution of the thrombomodulin C/T polymorphism in terms of CC, CT and CC/TT genotypes in CAD patients and controls were also presented. Chi-Square test and OR were used to analyze the distribution of the genotypic and allelic frequencies of the thrombomodulin C/T polymorphism in the cases and controls. All variables that were either statistically significant in the univariate analyses or potentially important with respect to prevention or biologically relevant variables were included in logistic-regression analyses. Final multivariable models were created through stepwise elimination of variables. Potential confounding was assessed with the use of multivariate models adjusted for participant’s characteristics and other major risk factors for coronary artery disease. All reported p values are two-tailed, with values less than 0.05 indicating statistical significance.

## RESULTS

In this un-matched case- control study, age was significantly associated with CAD (p-value <0.001). There were more males in the case group as compared to the control group (p-value <0.001). Whereas in the modifiable risk factors Body Mass Index (BMI) (p-value <0.001), Systolic blood pressure (SBP) (p-value <0.001), Diastolic blood pressure (DBP) (P-value 0.03), cholesterol (CHOL), Triglyceride (TG), High Density Lipoprotein (HDL) & Low density Lipoprotein (LDL) were significantly high in cases as compared to controls ((p-value <0.001) Table-I. Moreover, CAD group had a strong history of smoking (p-value <0.001), dyslipidemia (p-value 0.005) and hypertension (p-value <0.001), whereas, family history is non-significant among the groups (p-value 0.243) Table-I.

**Table-I:** Clinical and laboratory characteristics of patients with coronary artery disease and in the control Subjects.

Clinical Characteristics	Controls		Cases		p-value

	Mean	SD	Mean	SD	
Age	36.06	16.83	59.24	9.78	<0.001
BMI(kg/m^2^)	23.94	4.67	26.52	4.74	<0.001
SBP	122.05	13.49	130.67	18.74	<0.001
DBP	74.84	8.81	78.14	11.36	0.03
CHOL	165.20	33.62	198.18	49.35	<0.001
TG	122.54	72.06	163.16	56.51	<0.001
HDL	39.26	8.76	27.82	6.69	<0.001
LDL	112.21	30.16	125.31	36.17	<0.001

Gender *Frequency (%)*					
female	49(54.4)		20(23.9)		<0.001
male	41(45.6)		72(76.1)		

Family History	28 (31.1)		21 (22.8)		0.243
Smoking	10 (11.1)		31 (33.7)		<0.001
Dyslipidemia	17 (18.9)		35 (38.0)		0.005
Hypertension	06 (6.7)		54 (58.7)		<0.001

The genotyping frequencies of C1418T polymorphism in cases and control revealed that frequency of CC, CT and TT genotypes were 81(90%), 6(6.7%) and 3(3.3%) in controls and 67(72.8%), 20(21.7%) and 5(5.4%) in cases respectively. The frequency distribution of C and T alleles were 154(84%) and 30 (16%) in the CAD group and 168 (93%) and 12 (7%) in the control samples respectively. The difference between the two groups was statistically significant (p=0.04, χ^2^=3.96) having an OR of 2.5 (95% CI=1.001, 6.85) as presented in Table-II.

**Table-II:** Genotypic and allelic frequencies of the TM C/T polymorphism in CAD patients and Controls.

TM genotype	CAD cases (%)	Controls (%)	Total	p-value
CC	67(72.8%)	81(90%)	148	0.009*
CT	20(21.7%)	06(6.7%)	26	
TT	5(5.4%)	3(3.3 %)	8	
*TM allele*				
C	154(84)	168(93)	332	
T	30(16)	12(7)	42	0.04

χ^2^=3.96, df=1, OR=2.5 (95% CI= 1.001, 6.85),

* χ^2^p-value df= 2

The frequency distribution of CT and CT/TT showed a statistically significant association with CAD (p=0.003 & p=0.003 respectively) having an OR of 4.03 (95% CI=1.5, 10.6) and OR of 3.36 (95% CI=1.5, 7.7) respectively as shown in table-III.

**Table-III:** Genotypes comparison of C1418T of Thrombomodulin Gene in Patients with Coronary artery disease and in Control Subjects.

Genotype	Cases (%)	Controls (%)	Total	OR	95% CI	P value
CT	20(23)	6 (7)	26	4.03	1.5, 10.6	0.003
CC	67(77)	81 (93)	148			
Total	87	87	174			
TT	5 (7)	3 (4)	8	2.01	0.6, 3.6	0.472
CC	67 (93)	81 (96)	148			
Total	72	84	156			
CT/TT	25 (27)	9 (10)	34	3.36	1.5, 7.7	0.003
CC	67 (73)	81 (90)	148			
Total	92	90	182			

CC= Homozygous common genotype, CT= heterozygous genotype,

TT= Homozygous less frequent genotype.

The CAD samples were in Hardy-Weinberg equilibrium (HWE) with (χ^2^= 3.81, p>0.05) and control samples were also in HWE (χ^2^= 19.4, p>0.05).

Our multivariable logistic regression analysis (Table-IV) showed that besides the established risk factors such as increasing age, low levels of high density lipoprotein and male gender in Pakistani population, the CT/TT genotypes were an independent risk factor for the Coronary Artery Disease (CAD) in our sample. Study participants having CAD were 6.5 times more likely to exhibit a variant in cases than controls. The model explained 71.0% (Nagelkerke R2) of the variance in CAD and correctly classified 86% of cases.

**Table-IV:** Multivariable Logistic regression analysis showing risk factors for Coronary artery disease in a sample of Pakistani population.

Characteristics	Β	χ^2^	P	OR	95% Confidence Interval
Age	0.11	91.7	<0.001	1.1	1.07, 1.16
HDL	-0.16	35.1	<0.001	0.85	0.79, 0.91
Gender	1.18	6.05	0.021	3.27	1.19, 8.98
Genotype	1.87	6.1	0.03	6.5	1.25, 33.77

## DISCUSSION

Thrombomodulin is a glycoprotein present on the surface of endothelial cells.^13^ It has a role in vasoprotection by converting procoagulant thrombin into an anticoagulant.^6^ Based on the results of this un-matched case-control study, we found a significant association between C1418T polymorphism and CAD i.e. the frequency CT and TT genotypes are more in cases as compared to controls considering both cases and controls were in Hardy-Weinberg equilibrium. The findings of our study are in agreement with an Indian study conducted by Dogra et al^14^ which showed that TM C1418 T (Ala 455 Val) genotype was an independent risk predictor of Acute Myocardial Infarction (AMI) in young adults. The study also showed a strong association between TM 1418 C/T polymorphism (1418 CT + TT genotype) and smoking in the occurrence of AMI in young adults, whereas, among non-smokers patient, TM 1418 C/T polymorphism (1418 CT+ TT genotype) was not associated with AMI. A study conducted by Ranjith et al^15^ showed a positive association in a subgroup of smokers for the presence of T allele and myocardial infarction. A similar finding were also observed by shah et al^16^ where subjects with ≤49 years of age, the TM Ala 455 Val substitution was significantly higher in cases and thus increases the CAD risk by 3 folds. The results of our study was also supported by study conducted by Wu et al^10^ who showed that TM C1418T variant in American blacks increase the risk of ischemic heart disease by 6.1 folds, emphasizing the investigating genetic risk factor by ethnic origin. The possible explanation for these positive results could be that Ala 455 Val is present on 6 EGF- like domain which is important for anticoagulant effect of TM,^17^ thus variant in this region may leads to the generation of more thrombin which in turn leads to thrombosis. Apart from this, the conventional risk factors such as smoking, diabetes, hypertension, blood pressure, BMI and levels of serum cholesterol, LDL, HDL and triglycerides are also raised in cases when compared to controls.

In contrast to our study findings Van der Velden^5^ Ohlin and Marler^12^, Ireland et al^18^, Park et al^19^ and Chao et al^11^, who didn’t show any association between C1418T and CAD. While Shah^16^ et al were also unable to show any association between TM gene polymorphism with CAD of ages > 50 years of age in their study.

The functional basis for these diverse differences in the influence of C1418T polymorphism and CAD is unclear. The possible explanation for these contradictory results could be different geographical location, life style, dietary patterns and ethnicity. Therefore in the light of these observations the future studies should be conducted on a large scale comprising the different ethnic communities of Pakistan. The importance of identifying the mutation in different races is mentioned by Wu et al^10^ in their work that showed that mutation in Ala 455 Val in black increases the risk of CAD by 6 folds as compared to whites. The Ala455Val polymorphism also leads to stroke as mentioned by Cole et al^20^ in their study yet again emphasizing that mutation in this region leads to thrombotic occlusion.

### Limitations of the study

It includes small sample size. Apart from this we cannot exclude the presence of myocardial ischemia with conventional risk factors such as smoking, diabetes, hypertension in some of our control subjects. It may be possible that few of them may develop ischemia in future, thus follow up in these controls are required especially with C1418T polymorphism.

## CONCLUSION

TM C1418T polymorphism may be a risk marker in CAD patients in the population of Karachi, Pakistan.
